# A cluster analysis of attachment styles in patients with borderline personality disorder, bipolar disorder and ADHD

**DOI:** 10.1186/s40479-024-00271-2

**Published:** 2024-10-30

**Authors:** I. Kouros, M. Isaksson, L. Ekselius, M. Ramklint

**Affiliations:** 1 Department of Medical Science, Psychiatry, Uppsala University, Uppsala, Sweden; 2 Department of Women’s and Children’s Health, WOMHER, Uppsala University, Uppsala, Sweden

**Keywords:** Attachment, Attachment style questionnaire, Borderline personality disorder, Bipolar disorder, Attention-deficit/hyperactivity disorder (ADHD), Cluster analysis, Emotional dysregulation

## Abstract

**Background:**

Insecure adult attachment has been associated with psychiatric disorders characterized by emotional dysregulation, such as borderline personality disorder (BPD), bipolar disorder (BD) and attention deficit/hyperactivity disorder (ADHD). However, little is known about the differences in attachment patterns between these diagnostic groups. The aim of this study was to identify clusters of adult attachment style in a cross-diagnostic group of patients with BDP and/or BD and/or ADHD and explore the characteristics of these clusters based on temperament profile, childhood trauma and psychiatric diagnoses.

**Methods:**

K-means cluster analysis was used to identify subgroups, based on the Attachment Style Questionnaire Short Form dimensions, in a clinical cohort of 150 young adults (113 women and 37 men, mean age ± SD = 23.3 ± 2.1) diagnosed with BPD, and/or BD, and/or ADHD.

**Results:**

Three distinct clusters were identified: a *secure*, an *insecure/avoidant-anxious* and an *insecure/avoidant* cluster. These three clusters differed in temperament profile and related psychiatric diagnoses.

**Conclusions:**

The three clusters of attachment in individuals with BPD, BD and/or ADHD could support differentiation between the disorders as well provide information usable for planning of treatment.

## Background

Attachment theory, developed by John Bowlby and later empirically tested by Mary Ainsworth, describes how the interactions between a primary caregiver and a child form patterns for future relationships between the grown-up child and other individuals [1, 8]. The theory was initially based on observations of interactions between mothers and their children with the perspective that these interactions are an evolutionary adapted system that ensures a child’s survival through proximity to their caregiver [1, 8, 56]. It was early suggested by Bowlby that attachment-related experiences not only influence the development of internal working models of attachment for the child, but also have implications across the whole lifespan [9]. Hazan and Shaver theorized that, similar to the emotional bond that develops between a child and it’s caregiver, the same motivational system underlies the relationships that are shaped in adult life [34]. One of the models for conceptualizing adult attachment styles, which expands on the work of Bowlby, was proposed by Bartholomew and Horowitz. This model assumes that there are four typological styles of attachment: secure, preoccupied (anxious/ambivalent), fearful, and dismissing [5]. This typological approach implies that the attachment styles are mutually exclusive, however further research has promoted a more dimensional measure of adult attachment [29]. Based on a dimensional model of attachment patterns, Feeney and colleagues developed the attachment style questionnaire (ASQ) for the assessment of adults [26]. Feeney’s model proposes five dimensions of attachment: 1) Confidence that corresponds to secure attachment, 2) Need for Approval and 3) Preoccupation with Relationships that reflect insecure anxious attachment, and 4) Relationships as Secondary and 5) Discomfort with Closeness that describe insecure avoidant attachment. The ASQ has been studied in both clinical and non-clinical populations and has demonstrated good psychometric properties, supporting the measurement of attachment style in a dimensional way [4, 26, 28].

There is a substantial body of literature that has explored the association between insecure attachment and specific psychopathology, especially in disorders characterized by emotional dysregulation often associated with relational problems, such as attention-deficit/hyperactivity disorder (ADHD), bipolar disorder (BD), and borderline personality disorder (BPD) [30, 33, 55]. Although most studies assessing the association between attachment insecurity and ADHD focus on children, there is evidence to propose an association between insecure attachment in adulthood and ADHD. Storebo et al. [63], in their review concerning the association between insecure attachment and ADHD, argue that even if there is a relation between disorganized attachment and ADHD symptoms in children as well as in adults, it is unclear whether attachment insecurity leads to ADHD or the other way around. In contrast, the evidence for an association of insecure attachment with BD is limited. One study by Harnic et al. [33] showed a higher prevalence of insecure attachment style in patients with BD and cyclothymia compared to a control group. A meta-analysis by Herstell et all [35] that studied insecure attachment as a trans-diagnostic risk factor for BD, depression and schizophrenia, found a higher prevalence of insecure attachment in BD compared to healthy controls but there was no evidence of a difference in attachment patterns between the mental disorders. Finally, it has been proposed that the development of insecure attachment patterns may mediate the relationship between childhood trauma and the development of BPD later in life [30].

The categorical approach to mental disorders is, however, often problematic when it concerns disorders with overlapping symptoms such as ADHD, BD and BPD. High rates of comorbidity among them, around 20%, have also been reported, as well as implications of comorbidity’s role as a negative prognostic factor [3, 52]. Symptom overlap and high comorbidity between these disorders often complicates assessment and the diagnostic process in clinical practice with the risk of ending up with an inaccurate diagnosis. Moreover, the categorical nosology system in psychiatry has been criticized for high levels of heterogeneity and it has been suggested that a more dimensional approach might be more appropriate [37, 45]. One method that uses a dimensional approach is cluster analysis. Clustering is a method of stratification that sorts cases according to how similar they are to each other, and produces groups of cases that have more in common within the groups than between the groups, hence proposes homogenous subgroups based on multivariate observations and can be employed as a data exploration tool [64]. Cluster analysis allows the detection of characteristics shared by a group which leads to a better understanding of the unique combination of features that the group possesses [27]. Using this method offers an opportunity to explore the unique combination of attachment patterns of a patient group with overlapping symptoms beyond the diagnostic criteria and therefore the possibility to compare differences and similarities between groups.

BPD, BD, and ADHD are mental disorders that exhibit not only a considerable overlap of symptoms, but also of diagnostic criteria. For example, impulsivity is a core criterion in all three conditions and affective instability is a criterion in BD and BPD and, according to DSM-5, an associated feature of ADHD. Even though childhood trauma is more often recognized as a risk factor associated with BPD rather than with BD or ADHD, adverse events in childhood have been linked to all three diagnoses [22, 51]. The complexity of the disorders, and the challenges involved in establishing specific links between genetic and environmental factors and the diagnoses, has led many scientists to focus on traits of psychopathology instead of a specific diagnosis or diagnostic criteria. Adult attachment is one conceptual framework for the development of psychopathology that has received a lot of interest. There is also evidence suggesting that attachment patterns play an important role in the development of emotional dysregulation, a concept that is involved in the development of all three disorders [17, 49]. The association between attachment and risk factors for psychiatric disorders, such as emotional dysregulation, seems to be clearer than the association between insecure attachment and specific disorders [65]. Some studies suggest that insecure attachment plays a role in mediating the development of borderline features, but temporal causality has not been established [25, 58, 67]. Moreover, a combination of preoccupied and fearful attachment styles appears to be correlated to interpersonal dysfunction in BPD [13].

Originally, the bio-psycho-social-model was conceptualized for understanding development and course of both somatic and mental disorders in 1977 [24]. Comparatively, the biosocial theory of BPD was proposed by Linehan in 1993 [46]. According to this theory, the core dysfunction in persons with BPD can be located in their emotional regulation system; it is a result of interactions and transactions between biological vulnerabilities and invalidating environments during childhood. Crowell et al. [19] extended Linehan´s biosocial theory into the biosocial developmental model. This model considers impulsivity as a distinct developmental vulnerability and proposes that the trait impulsivity and trait emotional dysregulation though interactive may appear independently and influence personality development and functioning in different aspects [19]. In ADHD, trait impulsivity and trait emotional dysregulation are also considered inherited and influencing further development, and ADHD is a potential risk factor for development of BPD [22, 59]. In BD there is a lack of consensus concerning whether these traits are inherited and present before age of onset or not, but some authors have used the bio-psycho-social model exploring development of BD based on that assumption [41].

Based on the bio-psycho-social model for development of mental disorders, it could be assumed that temperament, the inherited part of personality traits is present at birth and constitute the biological basis for personality development. This will early influence relationships to caregivers and therefore have an impact on attachment development, and attachment patterns will further influence experiences in close relationships and therefore shape personality traits. Since attachment develops during the first years in life, and even if environmental factors including trauma might influence attachment it is assumed that the internal working models of attachment will remain. Attachment insecurity has been shown to partly explain the association between childhood trauma and personality development [60]. Interpersonal problems are supposed to be more common in BPD, thereafter in ADHD and less in BD [7, 21]. This could be explained using the bio-psycho-social model, assuming the interaction between temperament and early caregivers as more problematic in BPD and therefore resulting in more insecure attachment, forming the dysfunctional interpersonal interactions. In a previous study, our group explored the interaction between temperament and childhood trauma as part of this theoretical model of BPD development, and traumatic experiences in childhood were common but the BPD group differed very little from the others in this regard. The interaction between temperament and trauma had low explanatory power for a BPD diagnosis in this sample [42]. However, attachment was not included in the previous analyses, and based on the bio-psycho-social model, attachment patterns are likely to influence the development of BPD.

This explorative study aimed to identify subgroups of attachment patterns in a cross-diagnostic group of patients with BDP and/or BD and/or ADHD. Additionally, we wanted to assess whether the subgroups differed in temperament profile, frequency of childhood trauma, and comorbidity patterns. We hypothesized, based on the bio-psycho-social model and biosocial model of BPD development [19], that patients with a BPD diagnosis should be found more often in a cluster characterized by an insecure attachment profile and that this cluster would differ from the other clusters in presenting more maladaptive temperamental traits, more reported childhood trauma and more increased psychiatric comorbidity.

## Methods

### Participants

**Fig. 1:**
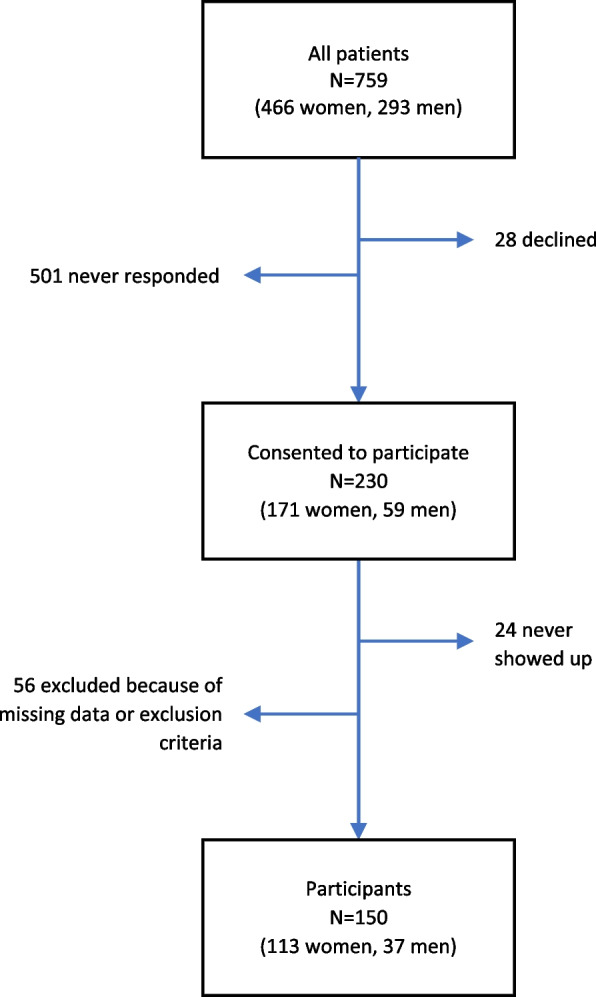
Flowchart of recruitment to the study

Patients who had been diagnosed with BPD, and/or ADHD, and/or BD, between May 1, 2005, and October 31, 2010, were recruited from an outpatient psychiatric clinic for young adults in Uppsala, Sweden. They were identified in the administrative patient register and sent a postal invitation to participate in the study, N = 759, mean age ± SD = 22.5 ± 2.7. Invitations were, for administrative reasons, sent to groups of patients at 24 different time-points from August 18, 2008 to May 13, 2011. Some patients were receiving ongoing psychiatric treatment at the clinic, others were not, and some had moved away and were living in other parts of the country. The aim was to include 200 participants, and inclusion stopped when in total, 230 (30%) individuals, 171 (22.5%) women and 59 (20.1%) men, had responded to the invitation. Of these, 29 did not attend the planned interview and 51 were later excluded either because they declined to participate in some parts of the study or due to missing data. The exclusion criteria included severe psychotic or manic symptoms at the time of the interview, and one patient was excluded because of current mania. A total of 150 individuals, 65.2% of those who agreed to participate, were included in this study. A flowchart of the recruitment process is presented in Fig. 1.

### Dropout analysis

Dropout analysis between the dropouts (n = 609) and the participants (*n* = 150) revealed that more women participated (61.4 vs 75.3%, *χ*^2^ = 15.320, *p* < 0.001), the study participants were older than the dropouts (22.5 vs 23.3, *F* = 11.494, p < 0.001), and fewer individuals with ADHD (43.1 vs 24.0%, *χ*^2^ = 27.763, *p* < 0.001), more individuals with BD (33.1 vs 40.0%, *χ*^2^ = 4.056, *p* = 0.044), and more individuals with some type of BPD/BD/ADHD comorbidity (12.6 vs 20.7%, *χ*^2^ = 10.879, *p* < 0.001) participated.

### Procedure

The study design was cross-sectional. The participants were interviewed by one psychiatrist (MR) and two residents in psychiatry (IK and NH), and they completed the questionnaires on one or two occasions, depending on the time needed. Social and demographic data were collected in an interview using a checklist. All BPD, BD and ADHD diagnoses were made through structured diagnostic interviews performed by MR, IK or NH, either at the clinic or as part of the study; all three are trained and quality-assured interviewers. The rate of agreement between interviewers is presented for each instrument below. Since inter-rater reliability was assessed repeatedly throughout the whole study period, the number of participating interviewers varied between occasions; the number of interviews and number of protocols for each instrument are therefore presented. The study was approved by the Uppsala University Ethics Committee, Dnr 2008/171.

## Assessments

### Structured clinical interview for DSM-*axis* I clinical version (SCID-I-CV)

SCID-I-CV [62] is a semi-structured clinical interview that assesses diagnoses according to the Diagnostic and Statistical Manual of Mental Disorders—fourth edition (DSM-IV). SCID-I-CV has shown high reliability for bipolar disorder [68]. The inter-rater reliability was assessed as prevalence-adjusted-bias-adjusted kappa (PABAK) [12]. The mean PABAK calculated for the three interviewers (MR, IK, NH) was 0.95 (range 0.91–0.97) based on six SCID-I-CV interviews (13 protocols). All participants who were diagnosed with BD type I, BD type II and BD not otherwise specified (NOS) were included in the BD group.

### Structured Clinical Interview for DSM-*axis* II (SCID-II)

SCID-II is a semi-structured diagnostic interview for the assessment of personality disorders according to the DSM-IV. The reliability of the SCID-II has been assessed in several studies. Although there is a high variation in the results, recent studies including a larger number of participants have reported higher reliability statistics with Cohen’s kappa for categorical assessment ranging from 0.48 to 0.98 [47, 48]. Participants completed the SCID-II-personality questionnaire. General personality disorder criteria and BPD criteria were assessed in all participants using the SCID-II interview. If the patient reported items above the cut-off for any other personality disorder, these disorders were evaluated through interviewing where all criteria for that disorder were assessed. There were no missing data for BPD but, in 13 cases, data were missing for other personality disorders. The inter-rater reliability calculated for the three interviewers (MR, IK, NH) was 0.85 (range 0.79–0.88) based on nine SCID-II interviews (23 protocols).

### Kiddie schedule for affective disorders and schizophrenia (K-SADS, Supplement for ADHD)

The K-SADS is a semi-structured interview for children aged between six and 18 years that measures current and past child psychiatric disorders according to the DSM-IV [39]. Due to the lack of validated interviews in Swedish for assessing ADHD in adults, the K-SADS supplement for ADHD was chosen for these young adults. The questions in the K-SADS supplement that corresponded to the DSM-IV criteria for ADHD were used. All criteria were assessed during the interview based on information obtained from the participants, however, without the presence of a parent. The participants were asked to consider whether they experienced symptoms before the age of seven years. The inter-rater reliability for the three interviewers (MR, IK, NH) was 0.72 (range 0.64–0.81) based on four interviews (11 protocols). Agreement between the interview results and self-reported symptoms of childhood ADHD, in this sample, has been previously reported [43].

### Attachment style questionnaire—short form (ASQ-SF)

The ASQ is a self-report questionnaire that is designed to measure adult attachment [26]. The ASQ-SF is the short form of the original ASQ and consists of twenty-nine items that derive from the forty items of the ASQ. These are rated on a 6-point Likert-type scale (1–6) where 1 corresponds to *totally disagree* and 6 to *totally agree*. The initial principal component analysis of the ASQ resulted in five dimensions: 1) Confidence (CO), which corresponds to secure attachment, 2) Need for Approval (NA) and 3) Preoccupation with Relationships (PR), which reflect insecure anxious attachment, and 4) Relationships as Secondary (RS) and 5) Discomfort with Closeness (DC), which describe insecure avoidant attachment. The ASQ-SF has been translated into Swedish and validated with internal consistency measures (Cronbach’s α) ranging from 0.62 to 0.78 [2, 32]. Compared to ASQ, the ASQ-SF has shown better psychometrics with Cronbach’s α ranging from 0.54 to 0.89 for different attachment dimensions [4, 38]. A confirmatory factor analysis of the ASQ-SF suggested a solution consisting of the five above mentioned subscales and also the broader constructs of anxiety (includes all items from NA and PR and half of CO), avoidance (includes all items from RS and DC and half of CO), and a response bias factor. One advantage of using the ASQ-SF in a population of young adults is that it is not dependent on experiences of romantic relationships as are other attachment questionnaires.

### Temperament and character inventory (TCI)

The TCI is a self-report questionnaire based on Cloninger’s psychobiological model [15]. The Swedish version of the TCI consists of 239 true/false items that measure temperament dimensions on four scales: novelty seeking (NS), harm avoidance (HA), reward dependence (RD), and persistence (P), and character on three scales: Self-Directedness, Cooperativeness, and Self-Transcendence. The psychometric properties of the Swedish version of the TCI appear to be similar to the English version with Cronbach’s α for the temperament scales ranging from 0.56 to 0.85 and test–retest correlations for the same scale ranging from 0.69 to 0.85 [10, 15]. Internal consistency for TCI in this material was 0.80 as determined by Cronbach’s alpha.

### Early trauma inventory self report – short form (ETISR-SF)

Originally developed by Bremner, the ETISR-SF is a self-report questionnaire that assesses childhood trauma based on four domains: general, physical, emotional, and sexual trauma [11]. These domains include eleven, five, five and six items, respectively. Both a total trauma score and a trauma score for each category can be measured. The psychometric properties of the Swedish translation of the ETISR-SF were similar to the original version with Cronbach’s α ranging between 0.74 and 0.76 for clinical groups and good discriminant validity when assessing the ETISR-SF total score (*z* = -6.796, *p* ≤ 0.001 [36].

### Statistical analysis

All analyses were performed using SPSS for Windows, version 28.0.1.0. Analysis of missing data showed that there were 0.18% (8 of 4350) of ASQ-SF values missing. Since this is a small proportion, we used expectation maximization to impute the missing values.

The clustering technique used was the K-means cluster analysis; the five subscales of the ASQ-SF, DC, RS, CO, NA and PR were used as determinants for the analysis. We conducted an elbow plot as part of the process of deciding the optimal number of clusters. Additionally, the clustering solutions for two to five clusters were tested since we assumed that a cluster solution with more than six clusters would result in clusters with a very low number of participants (less than 20). Using K-means clustering, significant effects can be detected even in small samples [20]. The validity of the cluster solutions was assessed using a line chart and cluster separation by performing one-way ANOVA with Tukey’s Honest Significance Difference post-hoc test using all five dimensions of the ASQ-SF. A criterion-related approach was used in order to explore the external validity of the clusters. We verified the stability of the cluster solution by repeating the K-means cluster analysis on a random split of half of the sample.

The analyses of the differences in the numerical variables between the clusters were calculated using one-way ANOVA with Tukey’s post-hoc test and the differences in categorical/grouping variables were calculated using the *χ*^2^-statistics and Bonferroni’s post-hoc test. Mean scores of all attachment dimensions was compared, using t-test, for participants with non-comorbid BPD (n = 19), ADHD (*n* = 20) and BD (*n* = 48).

## Results

**Fig. 2:**
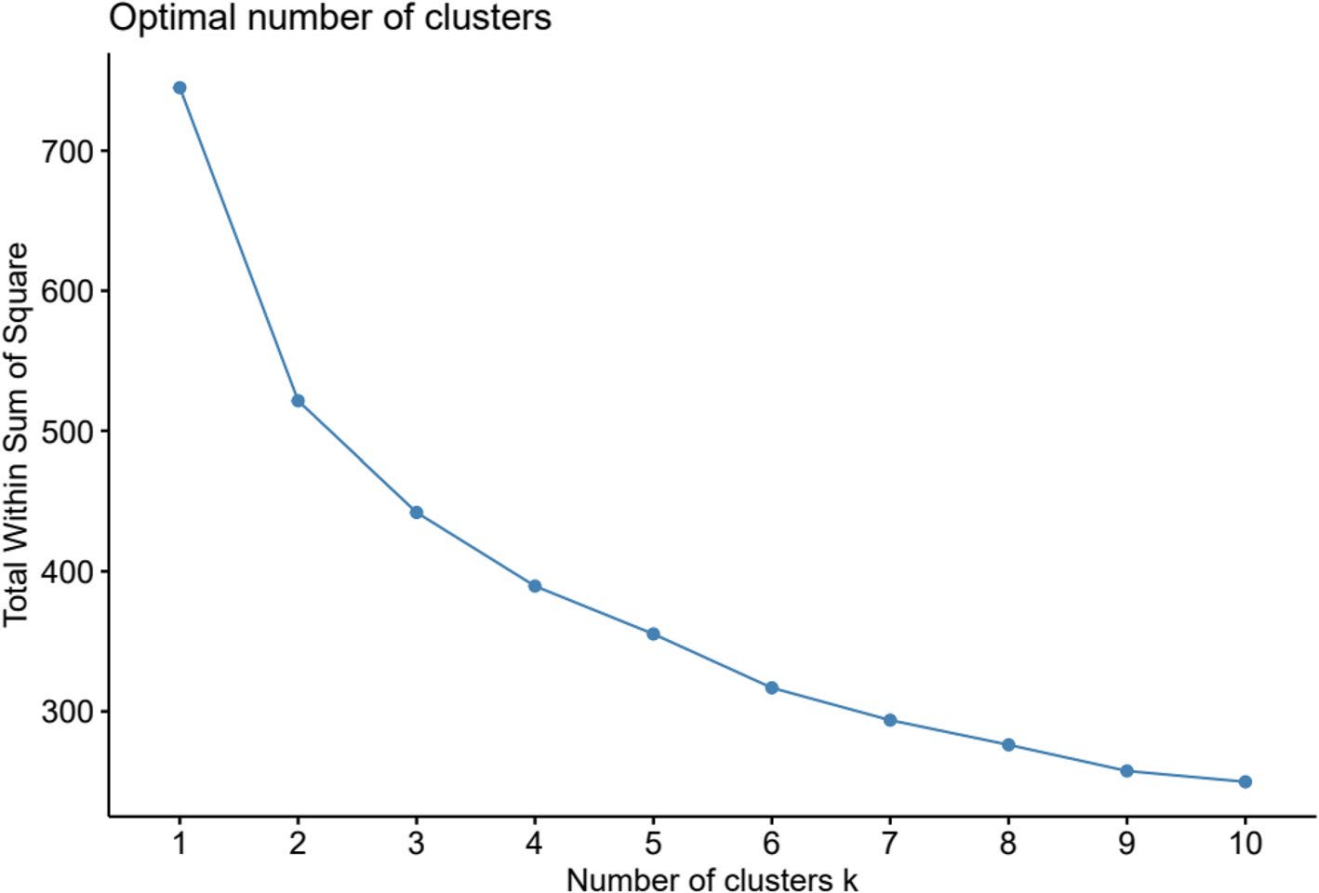
Elbow-plot for identification of the optimal number of clusters, however non-conclusive

The elbow plot did not reveal a clear elbow inflection point with either two or three clusters potentially being the optional number of clusters (Fig. 2). Since the k-means algorithm does not automatically propose a number of clusters, we tested different cluster solutions starting with two and up to five clusters. The two-cluster solution failed to show a significant difference in RS between the clusters and was therefore deemed unfit. Both the three and four-cluster solutions resulted in significant discrimination between the groups and with an acceptable number of participants in the clusters. The five-cluster solution resulted in a small group with 10 subjects and was therefore rejected. We evaluated the line charts for the three and four-cluster solutions in order to choose the one with the better fit. The post-hoc test of the three-cluster solution revealed significant differences in all dimensions between the three clusters, with the exception of DC between clusters one and two. The four-cluster solution showed significant similarities in all five dimensions between clusters in the post-hoc tests. Considering all analyses, the three-cluster solution was chosen as the best fit and with adequate separation between the clusters in the internal validation.

**Fig. 3:**
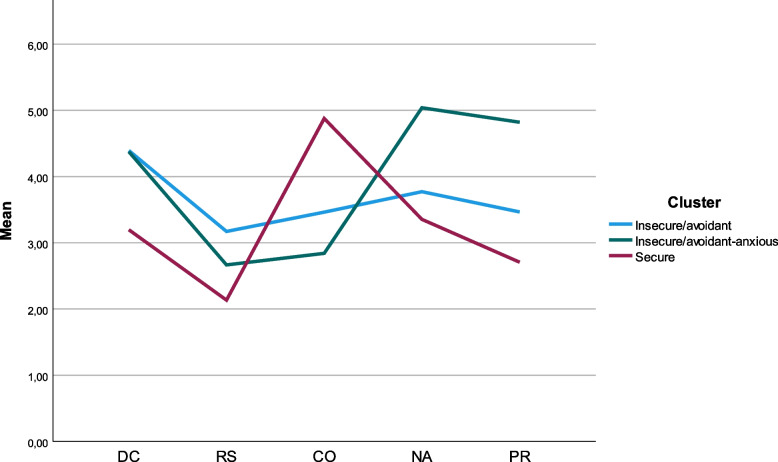
Line chart of the mean scores of all seven attachment dimensions DC, RS, CO, NA, PR (assessed by the ASQ-SF), presented for the three-cluster solution in 150 psychiatric patients diagnosed with ADHD and/or BPD and/or BP. The three clusters were called ‘Insecure/avoidant’, ‘Insecure/avoidant-anxious’ and ‘Secure’.DC: Discomfort with closeness, RS: Relationships as secondary, CO: Confidence, NA: Need for approval, PR: Preoccupation with relationships ASQ-SF: Attachment Style Questionnaire Short Form, ADHD: Attention Deficit Hyperactivity Disorder, BD: Bipolar disorder, BPD: Borderline personality disorder

**Fig. 4:**
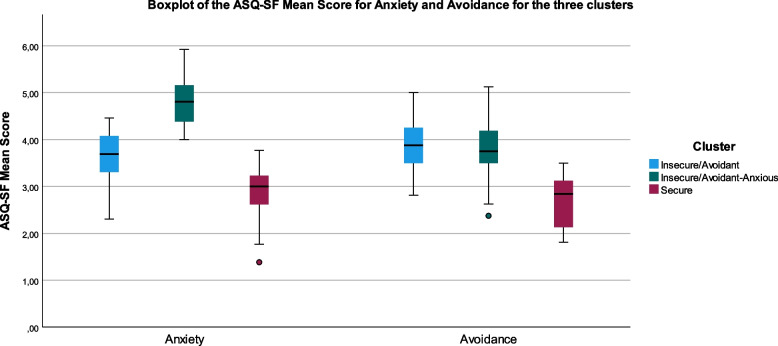
Visualization of the mean scores for the temperamental dimensions anxiety and avoidance*, presented for the three attachment clusters based on ASQ-SF, found in 150 young psychiatric out-patients diagnosed with ADHD, BPD and BD. ASQ-SF = Attachment Style Questionnaire-Short Form, ADHD: Attention Deficit Hyperactivity Disorder, BD: Bipolar disorder, BPD: Borderline personality disorder. *Anxiety and avoidance are two broader dimensions derived from ASQ. Anxiety includes all items from NA and PR and half of CO, avoidance includes all items from RS and DC and half of CO. (DC: Discomfort with closeness, RS: Relationships as secondary, CO: Confidence, NA: Need for approval, PR: Preoccupation with relationships)

The first cluster showed high scores for avoidance but intermediate scores in the anxiety dimensions and was therefore labelled *insecure/avoidant*, see Fig. 3 and 4, *n* = 58, mean (SD); CO = 3.46 (0.84), DC = 4.39 (0.67), RS = 3.17 (0.84), NA = 3.77 (0.83), PR = 3.47 (0.81), avoidance = 3.90 (0.50), anxiety = 3.64 (0.54). The second cluster displayed a low value in the CO dimension and high values in both avoidance and anxiety dimensions and was labelled *insecure/avoidant-anxious*, *n* = 66, CO = 2.84 (0.69), DC = 4.38 (0.79), RS = 2.67 (0.76), NA = 5.03 (0.56), PR = 4.82 (0.68), avoidance = 3.82 (0.58), anxiety = 4.85 (0.48). The third cluster had a higher mean score in the CO dimension and lower mean scores in all the other ASQ-SF dimensions and was therefore labelled *secure*, *n* = 26, CO = 4.88 (0.59), DC = 3.20 (0.75), RS = 2.13 (0.66), NA = 3.35 (0.84), PR = 2.70 (0.86), avoidance = 2.71 (0.53), anxiety = 2.84 (0.60) (Fig. 3 and 4).

Participants with BPD showed higher DC (M = 4.2, SD = 0.85) compared to participants with ADHD (M = 3.50, SD = 0.85, *p* = 0.014, Cohen’s d = 0.826), lower CO (M = 3.17, SD = 0.89 vs M = 4.16 SD = 0.87, *p* = 0.001, Cohen’s d = 1.128), higher NA (M = 4.71, SD = 0.79 vs M = 3.70, SD = 0.78, *p* =  < 0.001, Cohen’s d = 1.292), higher PR (M = 4.19, SD = 0.81 vs M = 3.30, SD = 0.99, *p* = 0.004, Cohen’s d = 0.979), higher Avoidance (M = 3.69, SD = 0.68 vs M = 3.1, SD = 0.68, *p* = 0.030, Cohen’s d = 0.722) and higher Anxiety (M = 4.41, SD = 0.68 vs M = 3.38, SD = 0.61, *p* =  < 0.001, Cohen’s d = 1.604). Between participants with BPD and BD, participants with BPD had higher NA (M = 4.71, SD = 0.79 vs M = 4.06, SD = 1.08, *p* = 0.020, Cohen’s d = 0.647).

There were no differences in gender distribution between the three clusters, with the first cluster including 41 females (70.7%), the second 53 (80.3%), and the third 19 (73.1%) (df = 2, *χ*^2^ = 1.622, p = 0.45).

The mean scores in all five dimensions of the full version of the ASQ for the three clusters were also calculated in order to compare them to the data from the Swedish validation study in healthy Swedish subjects (n = 1239) conducted by Andersson et al. [2]. The *secure* cluster showed similar scores in all dimensions to the mean scores of the healthy subjects in the Andersson et al. study while the *insecure anxious* cluster differed regarding DC and CO scores and the *insecure/avoidant-anxious* cluster regarding DC, CO, NA and PR scores. All three clusters showed similar scores in the RS dimension.

The mean scores of the five subscales of the three clusters were compared to the mean scores reported in the study of the psychometric properties of the ASQ-SF in pregnant women by Axfors et al. [4]. The *secure* cluster displayed slightly higher scores in DC, RS, NA and PR and slightly lower values in CO. The *insecure/avoidant-anxious* cluster showed higher values in DC, NA, PR, slightly elevated values in RS, and considerably lower values in CO, while the *insecure/avoidant* cluster displayed higher mean scores in DC and RS, lower in CO, and slightly higher in NA and PR.

The distribution of diagnoses of the three clusters is presented in Table 1. The *insecure/avoidant-anxious* cluster comprised significantly fewer individuals with ADHD than both the other clusters and more with BPD and BPD/BD comorbidity compared to the *insecure/avoidant* cluster.

**Table 1:** Distribution of ADHD, BPD, BD diagnoses between the three ASQ-SF cluster

	**Cluster**					
**Diagnosis**	***Insecure/*** ***avoidant*** (a) n (%)	***Insecure/*** ***avoidant-anxious*** (b) n (%)	***Secure*** (c) n (%)	***χ***^**2**^** (df = 2)**	***p***	***z-test***
Subclinical (*n* = 19)	7 (12.1%)	9 (13.6%)	3 (11.5%)	.105	.949	
ADHD (*n* = 20)	11 (19.0%)	1 (1.5%)	8 (30.8%)	16.410	< .001	a, c > b
BD (*n* = 48)	19 (32.8%)	19 (28.8%)	10 (38.5)	.827	.661	
BPD (*n* = 19)	4 (6.9%)	13 (19.7%)	2 (7.7%)	5.276	.072	
BPD with comorbidity (*n* = 35)	11 (19%)	23 (34.8%)	1 (3.8%)	12.877	.004	a, c < b
ADHD and BD (*n* = 9)	6 (10.3%)	1 (1.5%)	2 (7.7%)	4.427	.109	

*ASQ-SF* Attachment Style Questionnaire Short Form, *ADHD* Attention Deficit Hyperactivity Disorder, *BD* Bipolar disorder, *BPD* Borderline personality disorder

There were no differences observed between the three attachment clusters in terms of overall Axis-I comorbidity, the temperament dimensions of NS and PS, and frequency of childhood trauma. The *insecure/avoidant* cluster showed significantly lower scores in RD compared to the other clusters. The *secure* cluster showed lower mean scores in the HA dimension of the TCI, less Axis-II comorbidity and fewer individuals with any type of BPD/BD/ADHD comorbidity. All comparisons of the cluster characteristics are presented in Table 2.

**Table 2:** Description of differences in temperament (TCI), childhood trauma experiences (ETISR-SF), and comorbidity patterns between three clusters of attachment patterns based on ASQ-SF, found in 150 young psychiatric patients diagnosed with ADHD, BPD and BD

**Variables**	**ASQ-SF clusters**			**F ** **(df=2)**	***p*-value**	**Tukey HSD**
	**Cluster 1 ** ***insecure/*** ***avoidant*** **(a)**	**Cluster 2 ** ***insecure/ *** ***avoidant-anxious*** ***(b)***	**Cluster 3 ** ***Secure*** **(c)**			
**ASQ-SF-dimensions**^†^						
Avoidance (mean (SD))	3.9 (0.5)	3.8 (0.6)	2.7 (0.5)	48.256	<.001	a,b>c***
Anxiety (mean (SD))	3.6 (0.5)	4.8 (0.5)	2.8 (0.6)	162.682	<.001	b>a***>c***
**TCI**						
NS (mean (SD))	24.5 (6.1)	25.0 (5.6)	27.4 (6.2)	2.089	.13	
HA (mean (SD))	21.6 (6.5)	25.5 (5.4)	17.7 (5.7)	17.253	<.001	b>a*** a>c* b>c***
PS (mean (SD))	4.8 (1.7)	4.5 (2.0)	4.0 (1.9)	1.591	.2	
RD (mean (SD))	13.0 (3.2)	15.6 (2.9)	16.5 (2.6)	17.395	<.001	b,c>a***
**ETISR-SF**						
General trauma (mean (SD))	3.1 (2.0)	2.8 (2.0)	2.8 (1.8)	.308	.74	
Physical trauma (mean (SD))	1.5 (1.5)	1.6 (1.5)	1.0 (1.1)	1.461	.24	
Emotional trauma (mean (SD))	1.9 (1.9)	2.1 (1.8)	1.3 (1.8)	1.522	.22	
Sexual trauma (mean (SD))	1.0 (1.6)	1.1 (1.7)	0.9 (1.7)	.175	.84	
Total Trauma (mean (SD))	7.5 (5.0)	7.7 (4.6)	6.0 (4.0)	1.231	.30	
**Comorbidity**						
Sum of Axis-I Diagnoses (mean (SD))	2.5 (2.1)	2.6 (1.8)	1.9 (1.4)	1.293	.28	
Sum of Axis-II Diagnoses (mean (SD))	1.0 (1.4)	1.4 (1.3)	0.3 (0.6)	6.609	.002	b>c***

*ASQ-SF* Attachment Style Questionnaire-Short Form, *ETISR-SF* Early Trauma Inventory Self Report-Short Form, *TCI* Temperament and Character Inventory, *NS* novelty seeking, *HA* Harm avoidance, *PS* persistence, *RD* Reward dependence

^†^Anxiety and avoidance are two broader dimensions derived from ASQ. Anxiety includes all items from NA and PR and half of CO, avoidance includes all items from RS and DC and half of CO. (DC: Discomfort with closeness, RS: Relationships as secondary, CO: Confidence, NA: Need for approval, PR: Preoccupation with relationships)

*p<.05, *** *p*<.001

Post hoc for Avoidance: a>b, *p*=.001

Post hoc analysis for Anxiety: b>a, *p*=.001 b>c, *p*=.001 a>c, *p*=.001

## Discussion

The aim of this study was to explore potential subgroups of attachment patterns in individuals with BPD, and/or BD, and/or ADHD. We identified three separate clusters that differed in four of the five ASQ subscales. The three clusters were one with a secure attachment profile, one with a more insecure, avoidant and anxious profile, and one with an insecure and avoidant profile. Our original hypothesis was partially confirmed as the *insecure/avoidant-anxious* cluster was constituted by more patients with BPD comorbidity and differed from the other clusters in temperament profile. On the other hand, the *insecure/avoidant* cluster and the secure cluster did not differ in distribution of diagnoses or in frequency of reported childhood trauma.

Comparing the three clusters to previous studies in non-clinical populations supports the capability of ASQ to identify dimensions of attachment patterns in a clinical population, and that these dimensions have clinical relevance, since they differed regarding clinical characteristics. The presence of both anxiety and avoidance insecurity in one of the clusters also supports the hypothesis of attachment styles not being mutually exclusive [44].

The *secure* cluster was characterized by more confident individuals, who were less vulnerable and dismissing, having less focus on validation and reliance on others for fulfilling attachment needs, and showing resilience towards rejection and abandonment. The HA scores in the *secure* cluster were slightly higher than the mean HA scores in the Swedish population for the 20–35 year old group [10], but significantly lower than the scores for the other two clusters. In line with our results, one study in adolescents has shown that secure attachment is negatively correlated with HA [14].

The *insecure/avoidant-anxious* cluster displayed a fearful dismissive style of attachment with reliance on others, low self-esteem, and avoidance of intimacy. Attachment anxiety is related to negative working models of self, resulting in feelings of helplessness [31, 53]. Individuals in this cluster showed significantly higher scores for HA but no difference in scores for NS and PS, suggesting the importance of HA in the formation of attachment patterns in adulthood. This might indicate poorer emotional control and a higher dependence in social reward.

The *insecure/avoidant* cluster ASQ profile corresponds to a dismissing pattern of attachment where avoidance is related to a negative model of others. This involves difficulties in depending on others, feeling uncomfortable being close to others, and a higher degree of loneliness. This is supported by the temperament profile of this cluster, which showed lower RD scores than the other two clusters. Previous studies have shown RD to be positively associated with the ASQ dimension of Confidence and negatively associated with insecure avoidant attachment dimensions [50]. This cluster did not differ from the secure cluster concerning specific diagnoses.

Psychiatric patients have been shown to have more complex attachment patterns than non-clinical samples [28]. Severity of psychopathology has been shown to be related to more insecure attachment patterns and insecure attachment can be considered a general vulnerability for the development of mental disorders [54, 57]. There was a significant difference in the diagnoses of the participants in the *insecure/avoidant-anxious* cluster compared to the other two clusters since it consisted of more individuals with BPD comorbidity and fewer with ADHD. This suggests that attachment anxiety might play another role in the emotional dysregulation presented in BPD compared to ADHD. The differences in attachment patterns between patients with BPD and ADHD were also found when comparing mean values, showing a more insecure attachment style in participants with BPD without comorbidity compared to participants with an ADHD diagnosis without comorbid conditions.

Mikulincer et al. stated that there are other factors, such as temperament or a life history of trauma, that are more strongly correlated with the development of mental disorders than attachment patterns, but these factors can amplify the effects of the attachment experiences [54]. It is suggested that insecure attachment acts as a mediator in the development of BPD features in adulthood [6, 58]. The three clusters identified in our analysis did not, however, differ regarding the frequency of childhood trauma. Resilience to stress has been shown to negatively correlate with the temperament dimension HA and this could explain the development of more stress-enduring attachment patterns despite prior childhood trauma experiences in the *secure* cluster [40, 61, 66]. Furthermore, the development of secure internal working models and secure attachment to caregivers may buffer against the adverse effects of childhood trauma, as indicated in previous studies [18, 61]. The results illustrate the complex interaction between temperament, attachment patterns, and childhood trauma in psychiatric patients. Further studies including comparisons with non-clinical groups are important to disentangle the complex nature of these relationships.

As previous studies have shown, internal working models of attachment are susceptible to change and one way of achieving this is through specific psychological interventions [16, 23]. A focus on attachment-informed therapies for certain individuals may help the patients to better understand how interpersonal problems arise through understanding how they view themselves and others. Since the internal working models of attachment reflect the capability of building strong and meaningful relationships and influence interpersonal functioning, identifying individuals with insecure, and in particular avoidant, patterns might also explain the difficulties in adherence to therapeutic interventions in some cases.

This study has several limitations. There was a large dropout and more individuals with BD and fewer with ADHD agreed to participate, which might indicate sampling bias. On the other hand, a high percentage of individuals with comorbidity agreed to participate. Moreover, the dropout analysis was made based on age, sex and diagnoses. No other information was available for non-participants. The sample size was small; larger scale studies are therefore needed in the future to explore the validity of the clusters of patients with emotional dysregulation. All individuals in this study were young adult psychiatric patients, which limits the generalizability of our findings to older adult and non-clinical populations. Furthermore, the lack of instruments to measure emotional dysregulation and impulsivity is a limitation in this study and assessment of these symptoms could have further characterized the sample. One of the strengths of this study is that all individuals were thoroughly assessed in a clinical context by clinicians having high inter-rater reliability.

Even though attachments measures cannot be solely used to distinguish patients with BPD, ADHD and BD, our results suggest the presence of different patterns of attachment within these patients. Identifying patients with insecure adult attachment style might provide important information that could have implications for the therapeutic approach, since attachment patterns can change with treatment. To our knowledge, this is the first study that attempts to identify clusters of adult attachment styles in a cross-diagnostic group of patients diagnosed with different disorders with similar presentations including emotional dysregulation. Our hypotheses based on the bio-psycho-social model and biosocial model of BPD development [19], were partly confirmed and could be further explored in future studies.

## Conclusions

Three clusters based on attachment styles were identified in a group of individuals with BPD and/or BD and/or ADHD: a *secure* cluster, an *insecure/avoidant-anxious* and an *insecure/avoidant* cluster. These clusters differed according to diagnostic distribution and temperament profiles, but not trauma experience.

## Data Availability

No datasets were generated or analysed during the current study.

## Abbreviations

ADHD: Attention Deficit Hyperactivity Disorder
ASQ: Attachment Style Questionnaire
ASQ-SF: Attachment Style Questionnaire Short Form
BD: Bipolar Disorder
BPD: Borderline Personality Disorder
CO: Confidence
DC: Discomfort with Closeness
ETISR-SF: Early Trauma Inventory Self Report Short Form
HA: Harm Avoidance
K-SADS: Kiddie Schedule for Affective Disorders and Schizophrenia
NA: Need for Approval
NS: Novelty Seeking
P: Persistence
PR: Preoccupation with Relationships
RD: Reward Dependence
RS: Relationships as Secondary
SCID-I-CV: Structured Clinical Interview for DSM-IV Axis I– Clinical Version
SCID-II: Structured Clinical Interview for DSM-IV Axis II
TCI: Temperament and Character Inventory
